# The Relationship Between Illusory Health Beliefs, Recommended Health Behaviours, and Complementary and Alternative Medicine: An Investigation Across Multiple Time Points

**DOI:** 10.3390/bs15050614

**Published:** 2025-05-01

**Authors:** Andrew Denovan, Neil Dagnall, Kenneth G. Drinkwater

**Affiliations:** 1 School of Psychology, Liverpool John Moores University, Liverpool L3 3AF, UK; 2 Department of Psychology, Manchester Metropolitan University, Manchester M15 6BX, UK; n.dagnall@mmu.ac.uk (N.D.); k.drinkwater@mmu.ac.uk (K.G.D.)

**Keywords:** belief in science, complementary and alternative medicine, illusory health beliefs, illusory health beliefs scale, health locus of control, health outcomes

## Abstract

Illusory health beliefs (IHBs) represent invalid ideations about health and potentially impact health behaviours and practices in meaningful ways. Examples include the uptake of methods with less conclusive evidence/support (e.g., complementary and alternative medicine, CAM) versus empirically validated approaches (e.g., recommended by health professionals). However, measurement obfuscation of IHB has hindered construct operationalisation. This study examined a newly developed measure (the Illusory Health Beliefs Scale) in the context of health outcomes. Specifically, we explored adherence to recommended health behaviours (e.g., lifestyle, vaccines) and trust in healthcare professionals versus CAM use. Assessments included theoretically linked constructs, comprising health locus of control, belief in science, and belief in CAM. Using a sample of 1507 (734 males, 768 females, 7 non-binary), a statistical model tested relationships across time points. Path analysis revealed that IHBs aligned with openness to unorthodox treatments alongside lower confidence in conventional treatment methods. Crucially, locus of control and belief in science mediated/weakened this relationship, predicting greater adherence to health recommendations and trust in health professionals. Belief in CAM strengthened the relationship between IHBs and CAM use. The findings provide initial evidence regarding the contribution of IHBs to health outcomes, and a basis for future research to further explore the IHB-health relationship.

## 1. Introduction

Though scholars have failed to agree on a definition of paranormal belief (PB), delineations share the central notion that supernatural credence involves endorsement of phenomena that violate the basic principles of science (Dagnall et al., 2010; Drinkwater et al., 2018). Though lacking an empirical basis, PB remains common within general Western populations (Dagnall et al., 2024). Recognising that individuals typically capable of rational thought, critical evaluation, and reality testing paranormal endorse PB (Irwin, 2009; Dagnall et al., 2022; Drinkwater et al., 2024), psychological researchers explain credence in terms of cognitive and perceptual errors (e.g., reasoning misjudgements, Dagnall et al., 2014, 2016; biases, Williams et al., 2022; and non-clinical delusions, Irwin et al., 2012a, 2012b; Unterrassner et al., 2017). Commensurate with these elucidations, theorists regard the tendency to incorrectly attribute causation to unsubstantiated preternatural forces (e.g., luck), powers (e.g., mind reading), and entities (e.g., ghosts) as a fundamental aspect of PB (Irwin et al., 2013; Dagnall et al., 2017; Lange et al., 2019).

In the context of health, PB manifests as advocacy of irrational, ineffectually, and/or potentially detrimental notions related to wellbeing (i.e., self-serving illusions). These persist because they serve personal psychological functions such as sheltering individuals from reality (Schumaker, 1990) and affording emotional security (Irwin, 1993; Yarritu et al., 2015). Noting the potential adverse impact of supernatural credence on physical and mental welfare, Petrillo and Donizzetti (2012) operationalised paranormal health beliefs (PHBs) as supernatural convictions, that though lacking direct relationships with outcomes, influence wellbeing-related thoughts, choices, attitudes, and behaviours. Illustratively, superstitious individuals avoid or feel uncomfortable about attending medical appointments on dates they deem unlucky (Mjaess et al., 2021). Such choices illustrate why it is necessary for researchers to determine the extent, nature, and correlates of PHBs.

Cognizant of the importance of PHBS and the absence of an established measurement instrument, Petrillo and Donizzetti (2012) developed the Paranormal Health Beliefs Scale (PHBS), which is a 31-item measure that encompasses a range of domain content (i.e., magical and superstitious occurrences, religious convictions, mental powers, and faith in healers). Statistical evaluation found the PHBS was psychometrically sound (i.e., possessed a coherent factorial structure, adequate internal reliability, and discriminant and convergent validity) (Donizzetti & Petrillo, 2017). However, since their validation used only Italian participants and PHBS subject matter draws heavily on culture specific content (e.g., faith in saints) generalisability remained unattested. Additionally, Donizzetti and Petrillo (2017) and Petrillo and Donizzetti (2012) failed to assess important measurement properties (i.e., item difficulty, ceiling and floor effects, and response bias). The absence of these evaluations further restricted PHBS applicability.

Acknowledging the potential of the PHBS and the need for instrument development, Denovan et al. (2024b, 2024c) devised an adapted version of the measure for use with English-speaking samples. Modification included changing conceptual emphasis from paranormal to illusory health beliefs. This shift reflected the fact that supernatural ideation represents only a narrow subset of irrational wellbeing ideation. For example, endorsement of pseudoscience also influences judgments. Pseudoscientific belief denotes validation of non-scientific claims that purport to derive from scientific processes (Lobato & Zimmerman, 2018). Examples of pseudoscientific belief include complementary and alternative medicine (CAM), which are healthcare treatments that have developed outside of evidence-based medicine (Li et al., 2018). Alternative medicine is a replacement for evidence-based treatments, and complementary medicine exists alongside conventional treatment.

Through cognitive interviewing (i.e., think aloud protocol and concurrent probing), Denovan et al. (2024b) iteratively enhanced PHBS meaning and accessibility (i.e., revised culturally distinctive references, phraseology, and wording). Interviews concurrently revealed that a focus on illusory (rather than paranormal) health beliefs would ensure the scale captured a broader range of invalid ideations. In follow-up studies, Denovan et al. (2024a, 2024c) validated the emergent Illusory Health Beliefs Scale (IHBS) and tested factorial structure. The IHBS comprises six dimensions (Precognitive, Supernatural, Religious/Spiritual, Health Myths, Scepticism, and Health Pseudoscience), which possess satisfactory psychometric performance (i.e., internal reliability and validity). High positive correlations between Precognitive, Superstition, and Religious/Spiritual factors indicate that these subscales index overlapping, core facets of paranormal belief. Explicitly, convictions that psychic, preternatural (i.e., fate and destiny), and spiritual forces/powers influence health. These factors associate strongly with Health Myths, the tendency to endorsement wellbeing falsehoods.

Scepticism, the inclination to base health decisions on science and reject paranormal health beliefs, is weakly and inconsistently associated with other subscales (i.e., correlated negatively with Precognitive, Superstition, and Religious/Spiritual factors, and positively with Health Myths and Health Pseudoscience). Health Pseudoscience positively allies to other IHBS factors, suggesting that the construct embraces a combination of illusory ideations (i.e., paranormal, falsehoods, and misapplied science). Collectively, these produce views, which allude to science but concomitantly permit advocacy of the supernatural and fallacy. Recognising these features, Denovan et al. (2024a, 2024c) determined that the Health Pseudoscience subscale best functioned as a concurrent, standalone measure. Further analysis established that IHBS dimensions converged with theoretically related supernatural (e.g., paranormal belief) and health-based constructs (e.g., endorsement of CAM).

These studies indicate that illusory health beliefs are affiliated with/and can influence health attitudes and judgments (i.e., intention to adopt beneficial behaviours and treatment adherence) (Yarritu et al., 2015; Donizzetti & Petrillo, 2017; Donizzetti, 2018). Commensurate with this view, PB significantly predicts positive attitudes to pseudoscientific treatment (i.e., CAM) (Pettersen & Olsen, 2007). CAM endorsement is problematic because engagement with scientifically unattested treatments is common in the developed world (Tobbia et al., 2019).

Although CAM can on occasion provide relief (i.e., during palliative care, Gayatri et al., 2021) and prove harmless, generalised rejection of evidence-based approaches poses risks to health (e.g., vaccination uptake; Attwell et al., 2018). Hence, though IHBs may benefit individual wellbeing (e.g., increase optimistic bias; Makridakis & Moleskis, 2015) they typically facilitate poor decision making and outcomes.

For these reasons, it is vital that investigators develop an understanding of the ways that IHBs relate to mainstream healthcare use and adoption of scientifically recommended behaviours. Specifically, examination of the positive link between CAM affirmation and engagement (Lie & Boker, 2004) affords insights into the relationship between medical advice compliance and belief in science (Stosic et al., 2021).

Another pertinent variable is health locus of control (K. A. Wallston, 1992). High internal locus (belief in personal responsibility for wellbeing/illness) allies with greater engagement with medical advice (e.g., dietary habits; Marr & Wilcox, 2015). In contrast, external locus (belief that wellbeing is determined by powerful others or chance) predicts risky behaviours (Grotz et al., 2011). Consistent with these findings, Donizzetti and Petrillo (2017) reported a negative association between IHBs and internal locus, and a positive link with external locus. Correspondingly, external locus of control prognosticates higher levels of paranormal belief.

Therefore, research on locus of control affords insights into IHBs and their link with poor health outcomes. Moreover, it designates that individuals with greater levels of IHBs score higher on external health-related locus of control and engage less with health recommendations. However, the relationship between locus of control and health attitudes, intentions, and behaviours is complicated by the fact that engaging and adhering to scientific advice involves placing faith in medical professionals (e.g., greater confidence in practitioners facilitates stronger rapport and trust) (Brincks et al., 2010).

In this context, whilst immunisation provides defence against diseases some individuals refuse inoculation (Luyten & Beutels, 2016). Health-based locus of control (particularly external) predicts hesitancy. This relationship is stronger in individuals who possess illusory beliefs (e.g., religiosity) (Olagoke et al., 2021). Additionally, Aharon et al. (2018) reported that compliance with powerful others (e.g., doctors) positively correlated with vaccine compliance. Regarding CAM, the literature is mixed. Hence, it is unclear whether an internal or external locus of control best predicts use (Bishop et al., 2007).

### Current Study

This study, using statistical modelling, examined relationships between IHBs, adoption of recommended healthy behaviours, and CAM use. Modelling explored direct and indirect (mediating) relationships. Particularly, this assessed whether health-based locus of control, CAM advocacy (e.g., efficacy), and belief in science mediated the IHBs-health outcomes relationship. Alongside use of CAM, adherence to/compliance with recommended health behaviour assessment included adoption of health professional endorsed behaviours, confidence in health practitioners, and vaccine hesitancy.

## 2. Method

### 2.1. Participants

A sample of 1507 (*M*age = 52.93, range = 18 to 89) took part: 734 males (*M*age = 54.80, range = 21 to 87), 768 females (*M*age = 51.26, range = 18 to 89), and seven non-binary (*M*age = 41.00, range = 19 to 71). Participants completed measures three times, two months apart. Recruitment used Bilendi, an established supplier of quality panel data, which is equivalent to that collected using traditional approaches (Kees et al., 2017). The minimum age for recruitment was 18 years. The researchers specified a representative UK-based sample with a balance of genders and a broad age range.

### 2.2. Instruments

#### 2.2.1. Time Point One Measure

The Illusory Health Beliefs Scale (IHBS) (Denovan et al., 2024b, 2024c) assessed endorsement of scientifically unproven health beliefs (e.g., ‘Religious faith heals diseases’) at time point one (baseline). The instrument comprises 41-items and has a concurrent 10-item health pseudoscience scale (e.g., ‘Possessing a positive and optimistic attitude helps to prevent cancer’). Participants respond to statements using a five-point Likert scale (1 = Strongly disagree to 5 = Strongly agree). The IHBS has five subscales (Precognitive, Superstition, Religious/Spiritual, Health Myths, and Scepticism). The work of Petrillo and Donizzetti (2012), Fasce and Picό (2019), and Torres et al. (2020) informed these. IHBS reliability is good. Within this study, the researchers omitted the Scepticism subscale because it requires further psychometric development (Denovan et al., 2024c). In this study, Omega reliability was good/acceptable: Precognitive (*ω* = 0.91), Superstition (*ω* = 0.93), Religious/Spiritual (*ω* = 0.94), Health Myths (*ω* = 0.74), Health Pseudoscience (*ω* = 0.87).

#### 2.2.2. Time Point Two Measures

At time point two the researchers assessed the effect of mediator variables. This included health-based locus of control, belief in science, and belief in the veracity of CAM.

The Multidimensional Health Locus of Control Scale (MHLOC, B. S. Wallston et al., 1976; K. A. Wallston et al., 1978) assessed three health-based locus of control dimensions: internal, chance, and powerful others. MHLOC items appear as statements (e.g., ‘Having regular contact with my physician is the best way for me to avoid illness’) and participants record their responses on a six-point Likert scale (1 = Strongly disagree to 6 = Strongly agree). The MHLOC demonstrates good reliability (Moshki et al., 2007).

Additionally, the researchers included the six-item God Locus of Health Control (GodHLOC) Scale (K. A. Wallston et al., 1999), which used an identical response format to the MHLOC. This assesses the belief that God is responsible for health status (e.g., ‘God is in control of my health’). Scale reliability is good (Boyd & Wilcox, 2020). In this study, MHLOC (internal, *ω* = 0.77; chance, *ω* = 0.72; powerful others, *ω* = 0.79), and GodHLOC (*ω* = 0.98) demonstrated acceptable/good reliability.

The Belief in Science Scale (BIS, Farias et al., 2013) measured the degree to which respondents considered science a superior basis of knowledge. The instrument contains 10 items (e.g., ‘Science is the most efficient means of attaining truth’) and participants indicate their level of endorsement on a 6-point Likert scale (1 = *Strongly Disagree* to 6 = *Strongly Agree*). Reported internal reliability is high (Dagnall et al., 2019). In this study, reliability was excellent (*ω* = 0.93).

The Holistic Complementary and Alternative Medicines Questionnaire (HCAMQ, Hyland et al., 2003), using 11-items, assessed faith in CAM. Two dimensions concerning scientific veracity of CAM (CAM; five items), and holistic health (HH; six items) underpin items (e.g., ‘It is worthwhile trying complementary medicine before going to the doctor’). Participants respond using a six-point response scale (1 = Strongly agree to 6 = Strongly disagree). The HCAMQ has demonstrated adequate internal reliability (Aktaş & Bakan, 2021). This study Found satisfactory reliability: CAM (*ω* = 0.74), HH (*ω* = 0.82).

#### 2.2.3. Time Point Three Measures

At time point three health outcomes (adherence to recommended health behaviours, use of CAM, confidence in healthcare professionals, and vaccine hesitancy) were assessed alongside self-rated health status.

The Health Behaviour Inventory−20 (HBI−20, Levant et al., 2011) appraises adoption of health behaviours as recommended by health professionals, dimensions include: diet adherence, use of healthcare resources, anger and stress, preventive self-care, and substance use. Items appear as statements (e.g., ‘I limit the amount of salt I eat’) and participants respond via seven-point Likert-type scale (1 = never; 7 = always). Summing items produces a total score, which indicates adherence to and adoption of recommended health behaviours. The scale total has demonstrated acceptable reliability (α = 0.71; Levant et al., 2011). In this study, satisfactory HBI−20 reliability existed (*ω* = 0.75).

The Revised International Questionnaire to Measure Use of Complementary and Alternative Medicines (R-I-CAM-Q, Bryden & Browne, 2016) measures use of complementary and alternative medicine (CAM). The ten-item scale covers three areas: CAM provider used (e.g., Homoeopath), products (e.g., Herbal medicine), and self-help practices (e.g., Aromatherapy). Participants respond via three options: No = 0, Yes (Not in the last 12 months) = 1, Yes (In the last 12 months) = 2. Reported scale reliability is satisfactory (α = 0.74; Bryden & Browne, 2016). In this study, reliability was good (*ω* = 0.87).

A single item from the GP Patient Survey (Ipsos, 2024) measured confidence in healthcare professional (ConHP) (i.e., ‘During your last appointment with a healthcare professional, did you have confidence and trust in the healthcare professional you saw or spoke to?’). Participants responded via four options, 0 (‘I can’t remember, or it didn’t apply’) to 3 (‘Yes, definitely’).

The Adult Vaccine Hesitancy Scale (VHS, Luyten et al., 2019) assessed reluctance to engage in immunisation. The instrument comprises 10 items presented as statements (e.g., ‘Vaccines are important for my health’) and respondents record responses on a five-point Likert scale (1 = strongly disagree to 5 = strongly agree). Researchers report the VHS possesses satisfactory reliability (Shapiro et al., 2018). Reliability in this study was good (*ω* = 0.92).

Since self-rated health status influences treatment seeking and engagement in appropriate behaviour (K. A. Wallston, 1992) and potentially biases model estimation, the researchers controlled for self-rated health status in the statistical model. A single item, the Self-Rated Health Scale (SRH) assessed personal, perceived wellbeing (Meng et al., 2014). The SRH records participant responses on a slider scale, accompanied by the instructions: ‘please choose one point in this 0–100 scale, which can best represent your health today (0 means the worst and 100 means the best)’. Using a single item was efficient in terms of cost and brevity. Investigators report that the SRH performs comparably to longer health measures (Meng et al., 2014).

### 2.3. Procedure and Ethics

Participants completed the survey on three-time occasions two months apart. Prior to involvement, participants received information detailing procedures. Consenting individuals clicked a box indicating written consent. An ID number managed by BILENDI facilitated response matching (deleted after data collation). After completing the time point one/baseline measure (the IHBS), participants responded to MHLOC, BIS, and HCAMQ two months later (time point two). Participants subsequently completed the HBI−20, R-I-CAM-Q, VHS, and SRH at four months from baseline (time point three).

To limit order effects, questionnaire rotation occurred, using the in-built Qualtrics randomiser. Instructions told participants to complete the measures at their own pace, and no correct/incorrect responses existed. These remedies targeted evaluation apprehension and socially desirable responding. The Manchester Metropolitan University Ethics Committee (EthOS ID #52313) provided ethical approval.

### 2.4. Analysis Plan

Analysis comprised data screening, correlation inspection, and path model testing. The model assessed relationships between IHBs (as exogenous variables), health-based locus of control (InternalHLOC, ChanceHLOC, OthersHLOC, GodHLOC), belief in science (BISS), belief in CAM (CAM), and Holistic Health (HH) (mediators), and adherence to recommended health behaviours (HBI−20), use of CAM (CAM_USE), trust in Health Practitioner (HPConf), and vaccine hesitancy (VHS).

Path model evaluation employed Confirmatory Fit Index (CFI), Standardised Root-Mean-Square Residual (SRMR), and Root-Mean-Squared Error of Approximation (RMSEA). CFI > 0.95, SRMR < 0.05, and RMSEA < 0.05 signify good data fit (Hu & Bentler, 1999). Satorra–Bentler chi-square test (Asparouhov & Muthén, 2010) compared nested models. Computation of mediating relationships used bootstrapping with 95% bias-corrected confidence intervals (1000 resamples) (Preacher & Hayes, 2008).

## 3. Results

### 3.1. Data Screening and Correlations

Normality assessment found acceptable skewness (i.e., between −2.0 and +2.0), and kurtosis (i.e., between −7.0 and +7.0) (Hair et al., 2010). IHBs associated positively with health-based locus of control and CAM. Correspondingly, Precognitive, Superstition, and Religious/Spiritual correlated negatively with BISS. IHB subscales (except Superstition) correlated positively with HH. IHBs associated negatively with HPConf, positively with CAM_USE, VHS, and (weakly) with HBI−20. Associations were typically within the low-moderate interval (Table 1). The researchers interpreted correlational outcomes using the guidelines of Gignac and Szodorai (2016), which designate magnitudes as 0.10 (small), 0.20 (typical), and 0.30 (large).

### 3.2. Path Analysis

Path analysis found that the preliminary model (Model 1) exhibited good fit, χ^2^ (15, *N* = 1509) = 110.43, *p* < 0.001, CFI = 0.98, SRMR = 0.02, RMSEA = 0.06 (95% CI of 0.05 to 0.07). However, the scrutiny of relationships between exogenous and mediator variables showed instances of non-significance. Specifically, InternalHLOC with Illusory Health Beliefs (IHBs) apart from Health Pseudoscience, ChanceHLOC with Religious/Spiritual and Health Myths, OthersHLOC with Health Pseudoscience, GodHLOC with Health Myths, BISS with Precognitive and Health Myths, CAM with Superstition, and HH with Religious/Spiritual and Health Myths. Constraining these paths to zero (Model 2) produced good fit, χ^2^ (28, *N* = 1509) = 131.17, *p* < 0.001, CFI = 0.98, SRMR = 0.02, RMSEA = 0.04 (95% CI of 0.04 to 0.05).

**Figure 1:**
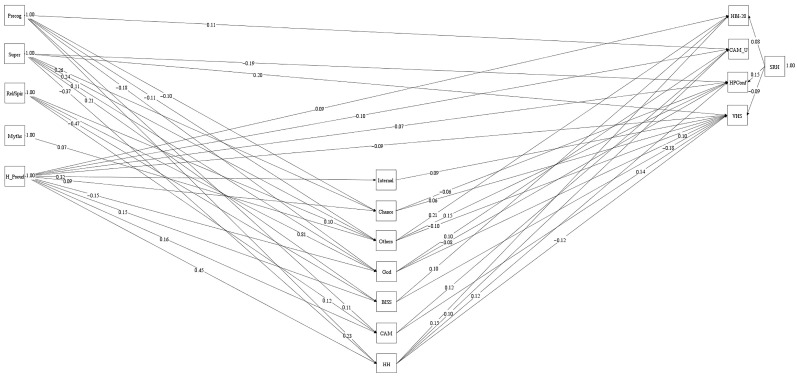
Multiple time point mediation model depicting putative relationships between illusory health beliefs, health-based locus of control, belief in science, beliefs about complementary and alternative medicine (CAM) and holistic health, health behaviour, use of CAM, confidence in healthcare professional, and vaccine hesitancy. Note. Self-reported health (SRH) is a covariate. Precog = Precognitive, Super = Superstition, (Rel/Spir = Religious/Spiritual, Myths = Health Myths, H_Pseud = Health Pseudoscience, Internal = Internal Health Locus of Control, Chance = Chance Health Locus of Control, Others = Others Health Locus of Control, God = God Health Locus of Control, BISS = Belief in Science, CAM = Belief in CAM, HH = Belief in Holistic Health, HBI−20 = Health Behaviour/Health Behaviour Inventory−20, CAM_U = Use of CAM, HPConf = Confidence in Healthcare Professional, VHS = Vaccine Hesitancy. Standardised regression weights between variables are shown. Error is not indicated but was specified for all variables. Only significant relationships at *p* < 0.05 are depicted to ease interpretation. Analysis used Bootstrapping significance estimates (1000 resamples).

Comparing Model 1 and 2 revealed non-significant worsening in fit, S–B *χ*^2^ = 20.86 (*df* = 13, *p* = 0.076), hence Model 2 was appropriate. However, Religious/Spiritual and Health Myths had no significant relationships with outcome variables. Specifying a model with these relationships constrained to zero (Model 3) produced good model fit, χ^2^ (36, *N* = 1509) = 141.32, *p* < 0.001, CFI = 0.98, SRMR = 0.03, RMSEA = 0.04 (95% CI of 0.03 to 0.05). Moreover, a non-significant worsening in fit existed, S–B *χ*^2^ = 9.79 (*df* = 8, *p* = 0.279). Relationship scrutiny revealed that Precognitive was a significant positive predictor of CAM_USE. Superstition predicted significantly lower HPConf, and higher VHS scores. Health Pseudoscience predicted significantly greater HBI−20, CAM_USE, ConHP scores, and lower VHS (Figure 1). SRH (as a covariate) significantly predicted greater HBI−20, HPConf, and lower VHS (no significant link existed with CAM_USE). Model 3 explained 12% of HBI−20, 17% of CAM_USE, 12% of HPConf, and 24% of VHS variance.

Mediation analyses revealed that OthersHLOC and HH positively mediated the Precognitive and HBI−20 relationship (Table 2). Regarding Superstition, OthersHLOC, BISS, and HH were positive mediators with HBI−20, as were BISS and HH regarding Health Pseudoscience and HBI−20. CAM and HH positively mediated, whereas GodHLOC weakened (negative mediator) the Precognitive-CAM_USE association. A similar result occurred for Superstition and Health Pseudoscience (albeit CAM was not significant for Superstition). OthersHLOC, GodHLOC, and HH positively mediated the Precognitive-HPConf relationship. ChanceHLOC, OthersHLOC, and HH enhanced the Superstition-HPConf link, and HH enhanced the Health Pseudoscience-HPConf affiliation. GodHLOC and HH weakened the link between Precognitive and VHS (CAM significantly increased this). ChanceHLOC, OthersHLOC, GodHLOC, BISS, and HH weakened the Superstition-VHS link. InternalHLOC, GodHLOC, BISS, and HH weakened the relationship between Health Pseudoscience and VHS (CAM enhanced this).

## 4. Discussion

IHBs correlated positively with health-based locus of control and CAM. At the subscale level, Precognitive, Superstition, and Religious/Spiritual associated negatively with Belief in Science and Precognitive, Religious/Spiritual, Myths, and Health Pseudoscience positively related to CAM. Regarding health-based locus of control, within the present study participants concurrently believed that internal (personal) and external (other: chance, powerful others, and God) factors influenced wellbeing. Although external influence implies loss of personal control, in some contexts it is desirable (Cheng et al., 2016). For example, individuals may lack self-control and be unable to quit unhealthy habits such as smoking. Hence, external support (e.g., support groups and therapy) is necessary. From this perspective, positive correlations between subscales specify that health locus of control is a complex construct, encompassing multiple sources of influence.

Additionally, the atypical positive association between internal and external locus of control dimensions likely reflects the generic definition of health employed in the present study. Since the researchers failed to specify health conditions, participant responses reflected subjective judgments based on myriad wellbeing contexts. Thus, while participants may feel able to fend off minor aliments by engaging in self-governed health practices, for other illnesses/conditions they believe the assistance of good fortune and assistance is required. This interpretation is consistent with K. A. Wallston (1992), who recommends that investigators consider health locus of control in conjunction with health value (Norman et al., 1998). Noting this, ensuing studies should include specific health concerns/conditions that vary in severity.

IHBs relationships with CAM (positive) and Belief in Science (negative) concurred with previous research (Van den Bulck & Custers, 2010; Denovan et al., 2024a). Collectively, these outcomes specify that presence of IBHs affiliates with greater openness to unorthodox remedies and reduced confidence in conventional treatments.

Regarding health behaviours, IHBs subscales correlated positively with scores on the Health Behaviour Inventory (HBI) (small) and Vaccine Hesitancy (typical, apart from Health Pseudoscience, which was small). Subscales, with the exception of Health Pseudoscience, were negatively (small) related to Confidence in Health Professionals. Finally, only Health Pseudoscience (small) was positively related to Self-Reported Health.

IHBS relationships with HBI and Vaccine Hesitancy were consistent with the belief-based findings reported earlier. Specifically, the notion that illusory beliefs reflect a generalised desire/anxiety to stay well/ward off illness by whatever means possible. Increased openness to alternative, unorthodox treatments and reduced faith in science/conventional medicine (i.e., immunisation and healthcare professionals) (see Horne & Weinman, 2002; Clarke & van Ameringen, 2015) typify this broadening focus.

This interpretation is consistent with Lazarus (1999), who described unorthodox beliefs as a coping mechanism for addressing emotional responses to health threats. Relatedly, Goode (2000) reported that increased exposure to health risks, especially those that caused anxiety, increased openness to alternative treatments and decreased trust in conventional medical practices. Indeed, patients with chronic illnesses demonstrate a broader focus. They seek out CAMs as a means of exerting control because they lack faith in conventional healthcare (Buchbinder & McGrail, 2005).

Findings from the predictive model indicated that greater belief in science, viewing powerful others (e.g., doctors, nurses) in control of health, and endorsing holistic beliefs about health enhanced the relationship between IHBs and adherence to recommended health behaviours. Additionally, greater endorsement of CAM and holistic health was affiliated with greater use of CAM in relation to IHBs. Belief in God as responsible for health lessened this. These findings make sense intuitively, because belief supportive of medical-based approaches (e.g., science, health professionals as responsible for health) predicted adherence to behaviour encouraged by medical evidence. Similarly, beliefs consistent with CAM predicted its use. Mozafari et al. (2024) reported stronger adherence to medical recommendations existed among individuals who viewed health professionals as the source of powerful others, and in turn better health outcomes. Additionally, Bauml et al. (2015) established that pro-CAM beliefs predicted significant variance in use of CAM.

In terms of Confidence in Healthcare Practitioners, an external locus of control regarding health (Others and God) enhanced this in response to IHBs (Chance lowered this). Although the finding regarding Others is consistent with prior evidence (e.g., Brincks et al., 2010), the result for God beliefs is more complex to disentangle. Regarding Chance, this is typically found to be negatively affiliated with trust in healthcare professionals. Explicitly, individuals with higher Chance beliefs do not characteristically view any individual/group as having control over their health, which can impede trust development (Brincks et al., 2010). Individuals who view God as responsible for health are more likely to place trust in God’s will than in the actions of health professionals (Rodriguez et al., 2023). However, trust in health professionals is complex, and affected by factors not modelled in this study, including relationship length, health professional characteristics, and presence/absence of illness (Brincks et al., 2010). Indeed, it is possible that trust in healthcare professionals exists among individuals with God beliefs, if they already possess strong relationships with their healthcare provider.

Lastly, health-based locus of control (apart from Chance) lessened the link between IHBs and vaccine hesitancy. Greater belief in science and endorsement of holistic views of health also had this effect, whereas Chance and endorsement of CAM enhanced vaccine hesitancy in relation to IHBs. These results align with Kouvari et al. (2022), who reported consistent relationships between Internal, Others (i.e., positive) and Chance (i.e., negative) with vaccine hesitancy. Moreover, an established literature base supports negative links between pro-CAM beliefs and increased vaccine hesitancy (c.f. Ward et al., 2023). Admittedly, this study did not examine critical contextual factors (e.g., types of CAM endorsed), which are important to consider given the relationship between CAM and the endorsement of scientific advice is multifaceted. Rather, this study indicates overall that IHBs aligned with openness to unorthodox remedies and reduced confidence in conventional treatments (e.g., immunisation and healthcare professionals), whereas locus of control and endorsement of science lessened this, predicting greater adherence to health recommendations/behaviours and trust in healthcare professionals.

### 4.1. Theoretical and Practical Implications

The current research was exploratory. However, assuming that IHBs function similarly to PBs (albeit for a health context) (Donizzetti & Petrillo, 2017; Donizzetti, 2018), theoretical connotations are possible. Specifically, IHBs within general populations epitomise self-serving delusions that influence convictions about personal welfare. These are unverified beliefs that safeguard emotional wellbeing (Irwin, 1993). These delusions are purported to be underpinned, in particular, by illusory control (Donizzetti & Petrillo, 2017; Donizzetti, 2018). For instance, the conviction that health can be influenced by actions/thoughts (of a paranormal nature, e.g., avoiding surgery on Friday 13th) that are not causally related to the outcome (preservation and/or promotion of health). This tendency is more likely to occur among individuals with a preference for experiential/intuitive thinking vs. analytical/rational thinking (from a dual-process perspective, specifically Cognitive-Experiential Self Theory; Epstein et al., 1996). Additional features of an experiential/intuitive thinking preference include less critical thinking engagement. These aspects can result in greater acceptance of unscientific claims and beliefs, typified within IHBs. Moreover, links with CAM can occur due to a need to establish control over health (e.g., during chronic illness) and greater reliance on intuition and personal experiences vs. objective data (Galbraith et al., 2018). This also explains weaker associations of IHBs with belief in science. Lack of perceived control attributes causation to external sources (e.g., fate), however illusion of control exhibits as internal locus of control (Irwin, 2000). Illusion occurs amongst individuals with a high need for control. For example, they desire to manage ill-health and not experience powerlessness. Therefore, high internal locus of control potentially indicates illusory control arising from low perceived ability to influence health (Denovan et al., 2024c). However, as emphasised, underpinning explanations for IHBs have yet to be empirically established. Crucially, this is an important avenue for future research.

Relationships of belief in science and locus of control with recommended health behaviours in the context of IHBs reflect the need to promote critical thinking and feelings of control in relation to health. For instance, developing strategies that promote public and patient education while encouraging evidence-based behaviours. Indeed, Wilson (2018) demonstrated that a science and critical thinking course reduced anomalous and pseudoscientific beliefs. Moreover, health professionals could help high IHB scorers develop feelings of control over health by emphasising patient involvement in decisions about their care (whilst considering their values, beliefs, and priorities), provide comprehensive information, choices and promote self-advocacy, support self-management via education/training, encourage self-care routines, health coaching, and address systemic issues (e.g., barriers to accessing healthcare) (Krist et al., 2017).

### 4.2. Limitations

Despite comprising multiple time points and access to a fairly large sample of balanced genders and age, this study contained limitations. A noteworthy issue is the lack of contextual factors with regard to studying belief in specific CAM treatments. Indeed, the present study utilised a general measure. Moreover, a similar concern existed for vaccine hesitancy. Ward et al. (2023) emphasised that views regarding vaccines varied greatly depending on the type of vaccine in question. Also, there exists a myriad of CAM treatments, with differing levels of perceived efficacy and subsequent endorsement. It would be useful for future researchers to study types of CAM and vaccine in relation to IHBs to add a more nuanced understanding of the predictive relationships established in this study.

An additional limitation relates to assessing health-based locus of control. Indeed, not specifying a health condition or conditions. This may have contributed to lack of clarity with regard to the links between this and IHBs. Lastly, the path analysis focused on the factor/scale level, rather than the item level. Although necessary due to the quantity of study variables, this may have impacted relationship precision due to (typically) lower reliability vs. single item indicators (Bollen & Pearl, 2013). Therefore, it would be useful for future research to corroborate the findings using an item level focus. To minimise variable burden, this could focus on testing distinct salient outcomes in separate investigations. For instance, testing the link between IHBs (Precognitive, Superstition, Health Pseudoscience), OthersHLOC, BIS, and vaccine hesitancy in one study, and examining the relationship between significant IHBs, CAM beliefs, and use of CAM in another. Lastly, a particular limitation relates to the fact that indirect explanations for IHBs exist, drawing on influences from the literature surrounding pseudoscience and paranormal belief. A critical step for future research thus includes testing the validity of dual-process and illusion of control explanations for IHB development and maintenance.

### 4.3. Conclusions

Overall, this was the first study to explore predictive and mediating relationships concerning IHBs and health outcomes. Critically, this research was exploratory and established that IHBs associated with openness to unorthodox treatments and lower confidence in conventional treatment approaches. However, locus of control and belief in science lessened/suppressed the intensity of this relationship and predicted stronger adherence to recommended health actions and trust in healthcare professionals. Although limitations existed, these findings provide a basis for future research to further explore the proposed mechanisms regarding IHBs and health outcomes.

## Figures and Tables

**Table 1:** Descriptive statistics and intercorrelations across study variables.

Variable	*Mean*	*SD*	1	2	3	4	5	6	7	8	9	10	11	12	13	14	15	16	17
Time 1																			
1 Precognitive	13.38	6.06		0.81 **	0.80 **	0.56 **	0.54 **	0.23 **	0.17 **	0.22 **	0.49 **	−0.12 **	0.26 **	0.19 **	0.09 **	0.34 **	−0.16 **	0.29 **	0.02
2 Superstition	17.22	7.84			0.79 **	0.62 **	0.45 **	0.20 **	0.23 **	0.29 **	0.54 **	−0.08 **	0.22 **	0.04	0.10 **	0.32 **	−0.22 **	0.33 **	−0.02
3 Religious Belief	21.54	9.95				0.59 **	0.51 **	0.22 **	0.20 **	0.27 **	0.68 **	−0.21 **	0.24 **	0.15 **	0.09 **	0.33 **	−0.15 **	0.29 **	0.02
4 Health Myths	15.61	4.72					0.55 **	0.23 **	0.19 **	0.25 **	0.40 **	−0.02	0.14 **	0.16 **	0.09 **	0.24 **	−0.11 **	0.20 **	0.02
5 Health Pseudoscience	31.78	7.45						0.33 **	0.17 **	0.18 **	0.22 **	0.02	0.24 **	0.41 **	0.19 **	0.31 **	0.01	0.06 *	0.08 **
Time 2																			
6 InternalHLOC	23.35	4.60							0.31 **	0.36 **	0.22 **	0.21 **	−0.06 *	0.47 **	0.20 **	0.17 **	0.04	0.01	0.21 **
7 ChanceHLOC	21.37	4.85								0.41 **	0.30 **	0.24 **	−0.17 **	0.30 **	0.13 **	0.10 **	−0.03	0.02	0.01
8 OthersHLOC	19.44	5.48									0.38 **	0.30 **	−0.18 **	0.21 **	0.27 **	0.15 **	0.07 *	−0.05 *	0.01
9 GodHLOC	13.14	8.27										−0.13 **	0.12 **	0.05	0.08 **	0.25 **	−0.15 **	0.26 **	0.03
10 Belief in Science	40.97	10.41											−0.43 **	0.28 **	0.20 **	0.01	0.15 **	−0.32 **	0.12 **
11 CAM	25.45	6.17												−0.20 **	−0.06 *	0.17 **	−0.14 **	0.30 **	−0.04
12 Holistic Health	22.05	4.37													0.24 **	0.17 **	0.16 **	−0.18 **	0.16 **
Time 3																			
13 HBI−20	79.34	15.97														0.27 **	0.20 **	−0.21 **	0.12 **
14 CAM_USE	3.10	3.51															−0.02	0.10 **	0.04
15 HPConf	2.55	0.62																−0.38 **	0.17 **
16 Vaccine Hesitancy	22.18	8.07																	−0.12 **
17 SRH	68.03	20.60																	

Note. Raw means are presented; CAM = Complementary and Alternative Medicine, HBI−20 = Health Behaviour Inventory−20, HPConf = Confidence in Health Professional, SRH = Self-Reported Health; * indicates *p* < 0.05, ** indicates *p* < 0.001.

**Table 2:** Specific indirect effects of illusory health beliefs on study outcomes through health-based locus of control, belief in science, and beliefs about complementary and alternative medicine and holistic health (for brevity, only significant indirect paths are displayed).

	HBI-20	CAM_USE	HPConf	VHS
Indirect path	*β* (95% CI)	*β* (95% CI)	*β* (95% CI)	*β* (95% CI)
Precognitive > OthersHLOC	0.02 * (0.01, 0.04)	-	0.02 * (0.01, 0.03)	-
Precognitive > GodHLOC	-	−0.02 * (−0.03, −0.01)	0.01 * (0.01, 0.03)	−0.02 * (−0.03, −0.01)
Precognitive > CAM	-	0.01 * (0.01, 0.03)	-	0.02 * (0.01, 0.03)
Precognitive > HH	0.04 ** (0.02, 0.05)	0.02 * (0.01, 0.04)	0.03 * (0.01, 0.04)	−0.03 * (−0.04, −0.01)
Superstition > ChanceHLOC	-	-	−0.02 * (−0.03, −0.01)	0.02 * (0.01, 0.03)
Superstition > OthersHLOC	0.05 ** (0.03, 0.08)	-	0.04 ** (0.02, 0.06)	−0.03 * (−0.04, −0.01)
Superstition > GodHLOC	-	−0.01 * (−0.02, −0.01)	-	−0.01 * (−0.02, −0.01)
Superstition > BIS	0.02 * (0.01, 0.04)	-	-	−0.04 ** (−0.05, −0.02)
Superstition > HH	0.06 ** (0.03, 0.07)	0.04 * (0.01, 0.06)	0.04 ** (0.02, 0.06)	−0.04 ** (−0.06, −0.02)
Health Pseudoscience > InternalHLOC	-	-	-	−0.03 * (−0.04, −0.01)
Health Pseudoscience > GodHLOC	-	−0.02 * (−0.03, −0.01)	0.01* (0.01, 0.02)	−0.02 * (−0.03, −0.01)
Health Pseudoscience > BIS	0.02 * (0.01, 0.03)	-	-	−0.03 ** (−0.04, −0.02)
Health Pseudoscience > CAM	-	0.02 ** (0.01, 0.03)	-	0.02 ** (0.01, 0.04)
Health Pseudoscience > HH	0.07 ** (0.04, 0.09)	0.04 * (0.02, 0.07)	0.05 ** (0.03, 0.08)	−0.05 ** (−0.08, −0.03)

Note. BIS = Belief in Science, CAM = Complementary and Alternative Medicine, HH = Holistic Health, HBI−20 = Health Behaviour Inventory−20, HPConf = Confidence in Health Professional, SRH = Self-Reported Health, VHS = Vaccine Hesitancy; * indicates *p* < 0.05, ** indicates *p* < 0.001 using Bootstrapping significance estimates (1000 resamples).

## Data Availability

This study was not preregistered. Data are accessible via FigShare: https://doi.org/10.6084/m9.figshare.28444610.

## References

[B1-behavsci-15-00614] Aharon A. A., Nehama H., Rishpon S., Baron-Epel O. (2018). A path analysis model suggesting the association between health locus of control and compliance with childhood vaccinations. Human Vaccines & Immunotherapeutics.

[B2-behavsci-15-00614] Aktaş B., Bakan A. B. (2021). Relationship between attitudes about medication adherence and complementary and alternative medicines in elderly individuals with chronic diseases. Alternative Therapies in Health & Medicine.

[B3-behavsci-15-00614] Asparouhov T., Muthén B. (2010). Computing the strictly positive Satorra-Bentler chi-square test in Mplus. Mplus Web Notes.

[B4-behavsci-15-00614] Attwell K., Ward P. R., Meyer S. B., Rokkas P. J., Leask J. (2018). “Do-it-yourself”: Vaccine rejection and complementary and alternative medicine (CAM). Social Science and Medicine.

[B5-behavsci-15-00614] Bauml J. M., Chokshi S., Schapira M. M., Im E. O., Li S. Q., Langer C. J., Ibrahim S. A., Mao J. J. (2015). Do attitudes and beliefs regarding complementary and alternative medicine impact its use among patients with cancer? A cross-sectional survey. Cancer.

[B6-behavsci-15-00614] Bishop F. L., Yardley L., Lewith G. T. (2007). A systematic review of beliefs involved in the use of complementary and alternative medicine. Journal of Health Psychology.

[B7-behavsci-15-00614] Bollen K. A., Pearl J., Morgan S. L. (2013). Eight myths about causality and structural equation modeling. Handbook of causal analysis for social research.

[B8-behavsci-15-00614] Boyd J. M., Wilcox S. (2020). Examining the relationship between health locus of control and god locus of health control: Is god an internal or external source?. Journal of Health Psychology.

[B9-behavsci-15-00614] Brincks A. M., Feaster D. J., Burns M. J., Mitrani V. B. (2010). The influence of health locus of control on the patient–provider relationship. Psychology, Health & Medicine.

[B10-behavsci-15-00614] Bryden G. M., Browne M. (2016). Development and evaluation of the RI-CAM-Q as a brief summative measure of CAM utilisation. Complementary Therapies in Medicine.

[B11-behavsci-15-00614] Buchbinder R., McGrail K. (2005). Beliefs and use of complementary and alternative medicine among patients with chronic illness. Psychology and Health.

[B12-behavsci-15-00614] Cheng C., Cheung M. W. L., Lo B. C. (2016). Relationship of health locus of control with specific health behaviours and global health appraisal: A meta-analysis and effects of moderators. Health Psychology Review.

[B13-behavsci-15-00614] Clarke A., van Ameringen M. (2015). The relationship between health anxiety and treatment-seeking behaviors. Journal of Health Psychology.

[B16-behavsci-15-00614] Dagnall N., Denovan A., Drinkwater K., Parker A., Clough P. (2017). Statistical bias and endorsement of conspiracy theories. Applied Cognitive Psychology.

[B14-behavsci-15-00614] Dagnall N., Denovan A., Drinkwater K. G., Escolà-Gascón Á. (2022). Paranormal belief and well-being: The moderating roles of transliminality and psychopathology-related facets. Frontiers in Psychology.

[B15-behavsci-15-00614] Dagnall N., Denovan A., Drinkwater K. G., Parker A. (2019). An evaluation of the belief in science scale. Frontiers in Psychology.

[B18-behavsci-15-00614] Dagnall N., Drinkwater K., Denovan A., Parker A., Rowley K. (2016). Misperception of chance, conjunction, framing effects and belief in the paranormal: A further evaluation. Applied Cognitive Psychology.

[B19-behavsci-15-00614] Dagnall N., Drinkwater K., Parker A., Rowley K. (2014). Misperception of chance, conjunction, belief in the paranormal and reality testing: A reappraisal. Applied Cognitive Psychology.

[B17-behavsci-15-00614] Dagnall N., Drinkwater K. G., Denovan A., Gascón A. E. (2024). Variations in positive well-being as a function of the interaction between paranormal belief and schizotypy. Frontiers in Psychology.

[B20-behavsci-15-00614] Dagnall N., Parker A., Munley G., Drinkwater K. (2010). Common paranormal belief dimensions. Journal of Scientific Exploration.

[B21-behavsci-15-00614] Denovan A., Dagnall N., Drinkwater K. G. (2024a). The illusory health beliefs scale: Validation using exploratory structural equation modelling and multidimensional Rasch analysis. Frontiers in Psychology.

[B22-behavsci-15-00614] Denovan A., Dagnall N., Drinkwater K. G. (2024b). The paranormal health beliefs scale: An evaluation using cognitive interviewing. Frontiers in Psychology.

[B23-behavsci-15-00614] Denovan A., Dagnall N., Drinkwater K. G., Gascón À. E. (2024c). The illusory health beliefs scale: Preliminary validation using exploratory factor and Rasch analysis. Frontiers in Psychology.

[B24-behavsci-15-00614] Donizzetti A. R. (2018). Paranormal health beliefs: Relations between social dominance orientation and mental illness. The Open Psychology Journal.

[B25-behavsci-15-00614] Donizzetti A. R., Petrillo G. (2017). Validation of the paranormal health beliefs scale for adults. Health Psychology Open.

[B26-behavsci-15-00614] Drinkwater K. G., Denovan A., Dagnall N. (2024). Paranormal belief, psychopathological symptoms, and well-being: Latent profile analysis and longitudinal assessment of relationships. PLoS ONE.

[B27-behavsci-15-00614] Drinkwater K. G., Denovan A., Dagnall N., Parker A. (2018). The Australian sheep-goat scale: An evaluation of factor structure and convergent validity. Frontiers in Psychology.

[B28-behavsci-15-00614] Epstein S., Pacini R., Denes-Raj V., Heier H. (1996). Individual differences in intuitive–experiential and analytical–rational thinking styles. Journal of Personality and Social Psychology.

[B29-behavsci-15-00614] Farias M., Newheiser A. K., Kahane G., de Toledo Z. (2013). Scientific faith: Belief in science increases in the face of stress and existential anxiety. Journal of Experimental Social Psychology.

[B30-behavsci-15-00614] Fasce A., Picó A. (2019). Conceptual foundations and validation of the pseudoscientific belief scale. Applied Cognitive Psychology.

[B31-behavsci-15-00614] Galbraith N., Moss T., Galbraith V., Purewal S. (2018). A systematic review of the traits and cognitions associated with use of and belief in complementary and alternative medicine (CAM). Psychology, Health & Medicine.

[B32-behavsci-15-00614] Gayatri D., Efremov L., Kantelhardt E. J., Mikolajczyk R. (2021). Quality of life of cancer patients at palliative care units in developing countries: Systematic review of the published literature. Quality of Life Research.

[B33-behavsci-15-00614] Gignac G. E., Szodorai E. T. (2016). Effect size guidelines for individual differences researchers. Personality and Individual Differences.

[B34-behavsci-15-00614] Goode E. (2000). Paranoia and the role of the media in the cultivation of beliefs in health risks. Social Science & Medicine.

[B35-behavsci-15-00614] Grotz M., Hapke U., Lampert T., Baumeister H. (2011). Health locus of control and health behaviour: Results from a nationally representative survey. Psychology, Health and Medicine.

[B36-behavsci-15-00614] Hair J. F., Black W. C., Babin B. J., Anderson R. E., Tatham R. L. (2010). Multivariate data analysis.

[B37-behavsci-15-00614] Horne R., Weinman J., Baum A., Singer J. (2002). Self-regulation and self-management in chronic illness. Handbook of psychology and health.

[B38-behavsci-15-00614] Hu L. T., Bentler P. M. (1999). Cutoff criteria for fit indexes in covariance structure analysis: Conventional criteria versus new alternatives. Structural Equation Modeling: A Multidisciplinary Journal.

[B39-behavsci-15-00614] Hyland M. E., Lewith G. T., Westoby C. (2003). Developing a measure of attitudes: The holistic complementary and alternative medicine questionnaire. Complementary Therapies in Medicine.

[B40-behavsci-15-00614] Ipsos (2024). GP patient survey 2024. *Ipsos*.

[B41-behavsci-15-00614] Irwin H. J. (1993). Belief in the paranormal: A review of the empirical literature. Journal of the American Society for Psychical Research.

[B42-behavsci-15-00614] Irwin H. J. (2000). Belief in the paranormal and a sense of control over life. European Journal of Parapsychology.

[B43-behavsci-15-00614] Irwin H. J. (2009). The psychology of paranormal belief: A researcher’s handbook.

[B44-behavsci-15-00614] Irwin H. J., Dagnall N., Drinkwater K. (2012a). Paranormal belief and biases in reasoning underlying the formation of delusions. Australian Journal of Parapsychology.

[B45-behavsci-15-00614] Irwin H. J., Dagnall N., Drinkwater K. (2012b). Paranormal beliefs and cognitive processes underlying the formation of delusions. Australian Journal of Parapsychology.

[B46-behavsci-15-00614] Irwin H. J., Dagnall N., Drinkwater K. (2013). Parapsychological experience as anomalous experience plus paranormal attribution: A questionnaire based on a new approach to measurement. Journal of Parapsychology.

[B47-behavsci-15-00614] Kees J., Berry C., Burton S., Sheehan K. (2017). An analysis of data quality: Professional panels, student subject pools, and Amazon’s mechanical turk. Journal of Advertising.

[B48-behavsci-15-00614] Kouvari K., Hadjikou A., Heraclidou I., Heraclides A. (2022). Health literacy, consciousness, and locus of control in relation to vaccine hesitancy and refusal. European Journal of Public Health.

[B49-behavsci-15-00614] Krist A. H., Tong S. T., Aycock R. A., Longo D. R. (2017). Engaging patients in decision-making and behavior change to promote prevention. Information Services & Use.

[B50-behavsci-15-00614] Lange R., Ross R. M., Dagnall N., Irwin H. J., Houran J., Drinkwater K. (2019). Anomalous experiences and paranormal attributions: Psychometric challenges in studying their measurement and relationship. Psychology of Consciousness: Theory, Research, and Practice.

[B51-behavsci-15-00614] Lazarus R. S. (1999). Stress and emotion: A new synthesis.

[B52-behavsci-15-00614] Levant R. F., Wimer D. J., Williams C. M. (2011). An evaluation of the Health Behavior Inventory−20 (HBI−20) and its relationships to masculinity and attitudes towards seeking psychological help among college men. Psychology of Men & Masculinity.

[B53-behavsci-15-00614] Li B., Forbes T. L., Byrne J. (2018). Integrative medicine or infiltrative pseudoscience?. The Surgeon.

[B54-behavsci-15-00614] Lie D., Boker J. (2004). Development and validation of the CAM Health Belief Questionnaire (CHBQ) and CAM use and attitudes amongst medical students. BMC Medical Education.

[B55-behavsci-15-00614] Lobato E. J. C., Zimmerman C., Kaufman A. B., Kaufman J. C. (2018). The psychology of (pseudo)science: Cognitive, social, and cultural factors. Pseudoscience: The conspiracy against science.

[B56-behavsci-15-00614] Luyten J., Beutels P. (2016). The social value of vaccination programs: Beyond cost-effectiveness. Health Affairs.

[B57-behavsci-15-00614] Luyten J., Bruyneel L., Van Hoek A. J. (2019). Assessing vaccine hesitancy in the UK population using a generalized vaccine hesitancy survey instrument. Vaccine.

[B58-behavsci-15-00614] Makridakis S., Moleskis A. (2015). The costs and benefits of positive illusions. Frontiers in Psychology.

[B59-behavsci-15-00614] Marr J., Wilcox S. (2015). Self-efficacy and social support mediate the relationship between internal health locus of control and health behaviors in college students. American Journal of Health Education.

[B60-behavsci-15-00614] Meng Q., Xie Z., Zhang T. (2014). A single-item self-rated health measure correlates with objective health status in the elderly: A survey in suburban Beijing. Frontiers In Public Health.

[B61-behavsci-15-00614] Mjaess G., Aoun F., Kazzi H., Karam A., Albisinni S., Roumeguère T. (2021). Myths, superstitions, and popular beliefs: Do they still impact our practice?. Annals of Surgery.

[B62-behavsci-15-00614] Moshki M., Ghofranipour F., Hajizadeh E., Azadfallah P. (2007). Validity and reliability of the multidimensional health locus of control scale for college students. BMC Public Health.

[B63-behavsci-15-00614] Mozafari S., Yang A., Talaei-Khoei J. (2024). Health locus of control and medical behavioral interventions: Systematic review and recommendations. Interactive Journal of Medical Research.

[B64-behavsci-15-00614] Norman P., Bennett P., Smith C., Murphy S. (1998). Health locus of control and health behaviour. Journal of Health Psychology.

[B65-behavsci-15-00614] Olagoke A. A., Olagoke O. O., Hughes A. M. (2021). Intention to vaccinate against the novel 2019 coronavirus disease: The role of health locus of control and religiosity. Journal of Religion and Health.

[B66-behavsci-15-00614] Petrillo G., Donizzetti A. R. (2012). Credenze illusorie sulla salute in adolescenza: Validazione di uno strumento di rilevazione. Giornale Italiano di Psicologia.

[B67-behavsci-15-00614] Pettersen S., Olsen R. V. (2007). Exploring predictors of health sciences students’ attitudes towards complementary-alternative medicine. Advances in Health Sciences Education.

[B68-behavsci-15-00614] Preacher K. J., Hayes A. F. (2008). Asymptotic and resampling strategies for assessing and comparing indirect effects in multiple mediator models. Behavior Research Methods.

[B69-behavsci-15-00614] Rodriguez A., Delbourgo Patton C., Stephenson-Hunter C. (2023). Impact of locus of control on patient–provider communication: A systematic review. Journal of Health Communication.

[B70-behavsci-15-00614] Schumaker J. F. (1990). Wings of illusion: The origin, nature, and future of paranormal belief.

[B71-behavsci-15-00614] Shapiro G. K., Tatar O., Dube E., Amsel R., Knauper B., Naz A., Perez S., Rosberger Z. (2018). The vaccine hesitancy scale: Psychometric properties and validation. Vaccine.

[B72-behavsci-15-00614] Stosic M. D., Helwig S., Ruben M. A. (2021). Greater belief in science predicts mask-wearing behavior during COVID-19. Personality and Individual Differences.

[B73-behavsci-15-00614] Tobbia I., Bella A., Safrany S., Greengrass C., Al Banna A., Mann-Isah N., Walsh P. (2019). Debating the merits and dangers of complementary and alternative medicine. Bahrain Medical Bulletin.

[B74-behavsci-15-00614] Torres M. N., Barberia I., Rodríguez-Ferreiro J. (2020). Causal illusion as a cognitive basis of pseudoscientific beliefs. British Journal of Psychology.

[B75-behavsci-15-00614] Unterrassner L., Wyss T. A., Wotruba D., Ajdacic-Gross V., Haker H., Rössler W. (2017). Psychotic-like experiences at the healthy end of the psychosis continuum. Frontiers in Psychology.

[B76-behavsci-15-00614] Van den Bulck J., Custers K. (2010). Belief in complementary and alternative medicine is related to age and paranormal beliefs in adults. European Journal of Public Health.

[B77-behavsci-15-00614] Wallston B. S., Wallston K. A., Kaplan G. D., Maides S. A. (1976). Development and validation of the Health Locus of Control (HLC) scale. Journal of Consulting and Clinical Psychology.

[B78-behavsci-15-00614] Wallston K. A. (1992). Hocus-pocus, the focus isn’t strictly on locus: Rotter’s social learning theory modified for health. Cognitive Therapy and Research.

[B79-behavsci-15-00614] Wallston K. A., Malcarne V. L., Flores L., Hansdottir I., Smith C. A., Stein M. J., Weisman M. H., Clements P. J. (1999). Does god determine your health? The god locus of health control scale. Cognitive Therapy and Research.

[B80-behavsci-15-00614] Wallston K. A., Strudler Wallston B., DeVellis R. (1978). Development of the Multidimensional Health Locus of Control (MHLC) scales. Health Education Monographs.

[B81-behavsci-15-00614] Ward J. K., Gauna F., Deml M. J., MacKendrick N., Peretti-Watel P. (2023). Diversity of attitudes towards complementary and alternative medicine (CAM) and vaccines: A representative cross-sectional study in France. Social Science & Medicine.

[B82-behavsci-15-00614] Williams C., Denovan A., Drinkwater K., Dagnall N. (2022). Thinking style and paranormal belief: The role of cognitive biases. Imagination, Cognition and Personality.

[B83-behavsci-15-00614] Wilson J. A. (2018). Reducing pseudoscientific and paranormal beliefs in university students through a course in science and critical thinking. Science & Education.

[B84-behavsci-15-00614] Yarritu I., Matute H., Luque D. (2015). The dark side of cognitive illusions: When an illusory belief interferes with the acquisition of evidence-based knowledge. British Journal of Psychology.

